# High Concentration of Low-Density Lipoprotein Results in Disturbances in Mitochondrial Transcription and Functionality in Endothelial Cells

**DOI:** 10.1155/2019/7976382

**Published:** 2019-06-10

**Authors:** Stefanie Gonnissen, Johannes Ptok, Christine Goy, Kirsten Jander, Philipp Jakobs, Olaf Eckermann, Wolfgang Kaisers, Florian von Ameln, Jörg Timm, Niloofar Ale-Agha, Judith Haendeler, Heiner Schaal, Joachim Altschmied

**Affiliations:** ^1^Heisenberg Group-Environmentally-Induced Cardiovascular Degeneration, IUF-Leibniz Research Institute for Environmental Medicine, 40225 Düsseldorf, Germany; ^2^Institute of Virology, Medical Faculty, Heinrich-Heine-University Düsseldorf, 40225 Düsseldorf, Germany; ^3^Core Unit Biosafety Level 2 Laboratory, IUF-Leibniz Research Institute for Environmental Medicine, 40225 Düsseldorf, Germany; ^4^Department of Anaesthesiology, HELIOS University Hospital Wuppertal, University of Witten/Herdecke, 42283 Wuppertal, Germany; ^5^Heisenberg Group-Environmentally-Induced Cardiovascular Degeneration, Central Institute of Clinical Chemistry and Laboratory Medicine, Medical Faculty, Heinrich-Heine-University Düsseldorf, 40225 Düsseldorf, Germany

## Abstract

Concentrations of low-density lipoprotein (LDL) above 0.8 mg/ml have been associated with increased risk for cardiovascular diseases and impaired endothelial functionality. Here, we demonstrate that high concentrations of LDL (1 mg/ml) decreased NOS3 protein and RNA levels in primary human endothelial cells. In addition, RNA sequencing data, in particular splice site usage analysis, showed a shift in NOS3 exon-exon junction reads towards those specifically assigned to nonfunctional transcript isoforms further diminishing the functional NOS3 levels. The reduction in NOS3 was accompanied by decreased migratory capacity, which depends on intact mitochondria and ATP formation. In line with these findings, we also observed a reduced ATP content. While mitochondrial mass was unaffected by high LDL, we found an increase in mitochondrial DNA copy number and mitochondrial RNA transcripts but decreased expression of nuclear genes coding for respiratory chain proteins. Therefore, high LDL treatment most likely results in an imbalance between respiratory chain complex proteins encoded in the mitochondria and in the nucleus resulting in impaired respiratory chain function explaining the reduction in ATP content. In conclusion, high LDL treatment leads to a decrease in active NOS3 and dysregulation of mitochondrial transcription, which is entailed by reduced ATP content and migratory capacity and thus, impairment of endothelial cell functionality.

## 1. Introduction

Diet plays a crucial role in the development and prevention of cardiovascular diseases. A diet high in saturated fat increases the risk of heart disease and stroke. It is estimated to cause about 31% of coronary heart disease and 11% of stroke worldwide. Cholesterol is carried through our blood by particles called lipoproteins: high-density lipoprotein (HDL) and low-density lipoprotein (LDL). HDL cholesterol reduces the risk of cardiovascular disease as it carries cholesterol away from the bloodstream. High levels of LDL cholesterol lead to atherosclerosis increasing the risk of heart attack and ischemic stroke. Already in 2002, Minamino et al. demonstrated that human atherosclerotic lesions contain vascular endothelial cells with senescence-associated phenotypes [1]. We have previously demonstrated for the first time that indeed high LDL is responsible for inducing senescence in human primary endothelial cells by treating them with 1 mg/ml LDL for one week. Those doses induced cellular senescence and loss of endothelial NO synthase (NOS3) [2]. Given the fact that endothelial functionality depends on the production of NO by NOS3 [3], it can be concluded that high doses of LDL lead not only to cellular senescence but also endothelial dysfunction, which is further characterized by endothelial activation, increased apoptosis sensitivity, and decreased migratory capacity [4, 5]. In agreement with our data in human primary endothelial cells, it was recently shown that intravenous injection of LDL in mice resulted in endothelial cell senescence and dysfunction *in vivo*. Interestingly, this dysfunction was accompanied by reduced mitochondrial oxygen consumption in the endothelium [6]. In this context, we already demonstrated that migratory capacity of endothelial cells depends on intact mitochondria. For that purpose, we had generated Rho^0^ human primary endothelial cells, which have nonfunctional mitochondria, such that their ATP production solely depends on glycolysis. We found that those cells are unable to migrate underscoring the importance of mitochondria as an energy source [7]. This is further emphasized by the fact that the inhibition of the mitochondrial ATP synthase by oligomycin in endothelial cells reduced the ATP content by approximately 60% [8]. Along the same lines, improvement of mitochondrial functionality with caffeine in endothelial cells increases ATP content and migratory capacity [9]. Thus, endothelial functionality depends on intact mitochondria and ATP produced therein. On the other hand, endothelial senescence and dysfunction seem to be accompanied by reduced mitochondria functionality. However, the underlying mechanisms are poorly understood and not well studied. Therefore, we investigated the effects of high LDL in human primary endothelial cells under conditions leading to endothelial dysfunction on mitochondrial DNA, RNA, protein levels, mass, and functionality.

## 2. Material and Methods

### 2.1. Cell Culture

Human primary endothelial cells were cultured in endothelial basal growth media (EBM) from Lonza, supplemented with hydrocortisone (1 *μ*g/ml), bovine brain extract (12 *μ*g/ml), gentamicin (50 *μ*g/ml), human epidermal growth factor (10 ng/ml), and 10% fetal calf serum (EBM complete). The cells were treated with high concentrations of LDL (high LDL) as previously described [2]. In detail, cells were seeded into cell culture dishes and incubated with the cultivation media for two days. After a medium change, the cells were further cultivated in the EBM complete medium (con) or the same medium containing 1 mg/ml LDL (high LDL). The media were changed every two days for 5 to 7 days depending on the experiment.

### 2.2. Isolation of Total RNA

For isolation of total RNA, cells were lysed using TRIzol (Thermo Fisher Scientific, Schwerte, Germany) and RNA was isolated according to the manufacturer's specifications. RNA was subjected to a second purification step using the RNeasy Mini Kit (Qiagen, Hilden, Germany). RNA concentrations were measured using a NanoDrop 2000c (Thermo Fisher Scientific, Schwerte, Germany), and RNA integrity was determined using a Bioanalyzer (Agilent, Waldbronn, Germany).

### 2.3. RNA Sequencing Analysis

RNA sequencing data was obtained from quadruplicate total RNA samples. After DNase treatment, a library for sequencing was constructed using the TruSeq® Stranded mRNA Sample Preparation kit (Illumina), according to the Ribo-Zero protocol to remove ribosomal RNA. Subsequently, the libraries were sequenced using HiSeq3000 (Illumina) generating an average of 392 million single-end reads per sample. Library constructions and sequencing were performed at the Genomics and Transcriptomics Laboratory at the Biological Medical Research Centre (BMFZ) of the Heinrich-Heine University Düsseldorf. The quality of the reads was assessed using the tool FASTQC by Andrews (http://www.bioinformatics.babraham.ac.uk/projects/fastqc/) and MultiQC [10]. Subsequently, with the help of Trimmomatic version 0.36 [11], reads were trimmed or discarded based on their base calling quality and their adapter content. Afterwards, the extent of rRNA depletion was measured by mapping the reads to rRNA databases using the SortMeRNA algorithm version 2.1b [12]. For alignment and the following analyses, the human genomic reference sequence (GRCh38) and annotation data (release 91) were downloaded from Ensembl [13] and BioMart [14]. After aligning the reads to the human reference using the two-pass mapping protocol of the STAR aligner (2.5.4b) [15], expression of the mitochondrial genes was calculated with the HTSeq python script [16]. Read coverage of gene-to-gene boundaries of mitochondrial transcripts was calculated using the SAMtools software package [17]. For DGE analysis with the R package DESeq2 version 1.18.1 [18], count matrices were generated using the software salmon version 0.9.1 [19].

Scripts used for this work are publicly available at https://github.com/caggtaagtat/Endothelial-mitochondria. FASTQ file preparation and alignment was accomplished using custom BASH shell scripts in the environment of the High Performing Cluster of the Heinrich-Heine University Düsseldorf. Computational support and infrastructure were provided by the “Centre for Information and Media Technology” (ZIM) at the Heinrich-Heine-University Düsseldorf.

### 2.4. Real-Time PCR Analysis of Transcript Levels

Total RNA was treated with RNAse-free DNase and reversed transcribed using SuperScript IV (Thermo Fisher Scientific, Schwerte, Germany) with random hexamers (pdN_6_) and oligo dT_20_ as primers. Relative transcript levels were determined by semiquantitative real-time PCR using the nuclear-encoded transcript for the ribosomal protein L32 (RPL32) as reference. The PCR reactions were done in a Rotor-Gene Q instrument (Qiagen, Hilden, Germany) using the SYBR Green qPCR Mastermix (Bimake, Munich, Germany) with the primer pairs listed below. All primer pairs for the analysis of nuclear transcripts were intron-spanning. For quantitation of mitochondrial transcripts, control reactions were performed with mock cDNAs, which were generated in a cDNA synthesis reaction without SuperScript IV. Relative expression was calculated as 2^−ΔCt (Ct gene of interest–Ct RPL32)^. The following primer pairs were used: MT-ND2: 5′-TCATAGCAGGCAGTTGAGGTG-3′/5′-CGTGGTGCTGGAGTTTAAGTTG-3′, MT-CYB: 5′-CATCGGCATTATCCTCCTGCT-3′/5′-ATCGTGTGAGGGTGGGACTG-3′, MT-CO3: 5′-AGGCATCACCCCGCTAAATC-3′/5′-ACTCTGAGGCTTGTAGGAGG-3′, MT-RNR1: 5′-CAAAACTGCTCGCCAGAACAC-3′/5′-GAGCAAGAGGTGGTGAGGTTG-3′, unprocessed mtRNA precursor transcripts: 5′-CGGACTACAACCACGACCAA-3′/5′-CCAAGGAGTGAGCCGAAGTT-3′(region 1) and 5′-AGAGGCCTAACCCCTGTCTT-3′/5′-TGCCTAGGACTCCAGCTCAT-3′ (region 2), TFAM: 5′-GATTCACCGCAGGAAAAGCTG-3′/5′-GTGCGACGTAGAAGATCCTTTC-3′, TFB1M: 5′-AGTGGCAGAGAGACTTGCAG-3′/5′-TTCCACCAGCTTGAATGGCT-3′, TFB2M: 5′-GCTGGAAAACCCAAAGCGTA-3′/5′-GTCTATTACAGTGGCGCTGC-3′, and RPL32: 5′-GTGAAGCCCAAGATCGTCAA-3′/5′-TTGTTGCACATCAGCAGCAC-3′.

### 2.5. Determination of Mitochondrial DNA (mtDNA) Content

Total DNA was isolated using the QIAamp DNA Mini Kit (Qiagen, Hilden, Germany). DNA concentrations were measured using a NanoDrop 2000c (Thermo Fisher Scientific, Schwerte, Germany). Relative mtDNA levels were determined by semiquantitative real-time PCR using the single copy nuclear nucleoredoxin (NXN) gene as reference. PCR reactions were done in a Rotor-Gene Q instrument (Qiagen, Hilden, Germany) using the SYBR Green qPCR Mastermix (Bimake, Munich, Germany) with the primer pairs listed below. Relative mtDNA content was calculated as 2^−ΔCt (Ct mtDNA–Ct NXN)^. The following primer pairs were used: D-loop: 5′-AGCCACTTTCCACACAGACATCAT-3′/5′-ATCTGGTTAGGCTGGTGTTAGGGT-3′ and NXN: 5′-CCACTCTTGTGTTCTCAGGCAGG-3′/5′-CGTGGGAGCTGTTTGTATGATATGAACC-3′.

### 2.6. Immunoblotting

Cells were lysed with JNK buffer (10 mM Tris-HCl, pH 7.5,150 mM NaCl, 2.5 mM KCl, 0.5% (*v*/*v*) Triton X-100, 0.5% (*v*/*v*) IGEPAL CA-630), and proteins were separated by SDS-PAGE followed by immunoblotting using primary antibodies against NOS3 (1 : 500, BD, Heidelberg, Germany) and ERK 1/2 (1 : 2000, Cell Signaling Technology, Frankfurt, Germany). All antibodies were diluted in 1% nonfat dry milk. Primary antibodies were incubated overnight at 4°C followed by HRP-coupled secondary antibodies for 2 h at room temperature. For protein detection, the Pierce™ ECL Western Blotting Substrate (Thermo Fisher Scientific, Schwerte, Germany) was used. Semiquantitative analysis from scanned blots was performed by using ImageJ 1.46r [20].

### 2.7. Cell Migration Assay

Cell migration assay was performed as described previously [9]. Wound closure was determined by setting a small wound and measuring wound width directly afterwards and 2 h later. Pictures were acquired with the Axiovert 200M fluorescent microscope from Carl Zeiss (Jena, Germany).

### 2.8. ATP Assay

Cells were lysed with JNK buffer, and ATP was measured in total lysates as described previously [9].

### 2.9. Mitochondrial Mass

Cells were treated with nonyl acridine orange and measured by flow cytometry as previously described [21].

### 2.10. Statistics

Data were analyzed using unpaired Student's *t*-tests. Differences in gene expression across samples were calculated using the Wald test of the R package DESeq2. *p* values were adjusted to the number of Wald tests following the Benjamini-Hochberg procedure [18]. Adjusted *p* values lower than 0.05 were considered significant.

## 3. Results

### 3.1. High LDL Induces Loss of NOS3 Protein and Overall NOS3 mRNA Levels Accompanied by Shifting Alternative Splice Site Use towards Inactive NOS3 Transcript Isoforms

**Figure 1:**
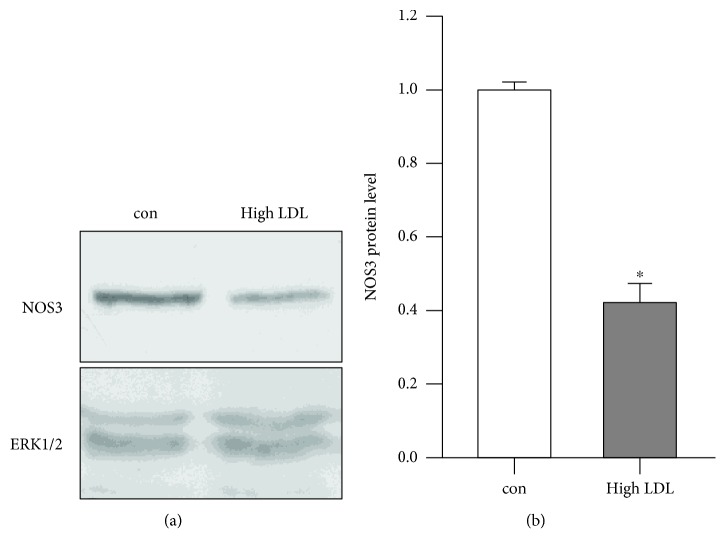
High LDL decreases NOS3 protein levels. Human primary endothelial cells were cultured in the standard medium (con) or medium containing 1 mg/ml LDL (high LDL) for 7 days. Full-length NOS3 protein was detected by immunoblot; ERK1/2 served as a loading control. (a) Representative immunoblots. (b) Semiquantitative analysis. Data are mean ± SEM; *n* = 7; *p* < 0.05 vs. con.

High LDL importantly contributes to the development and progression of cardiovascular diseases. However, the underlying molecular mechanisms how high LDL influences endothelial cell functionality are as yet poorly understood. In particular, detailed transcriptome analyses including the mitochondrial transcripts have not been addressed before. Therefore, we performed RNA deep sequencing of human primary endothelial cells after 7 days of treatment with high LDL. Prior to RNA sequencing, however, we validated that also in the current experimental setting, treatment with high LDL led to a reduction in NOS3 protein levels as described previously by us [2] (Figures 1(a) and 1(b)).

**Figure 2:**
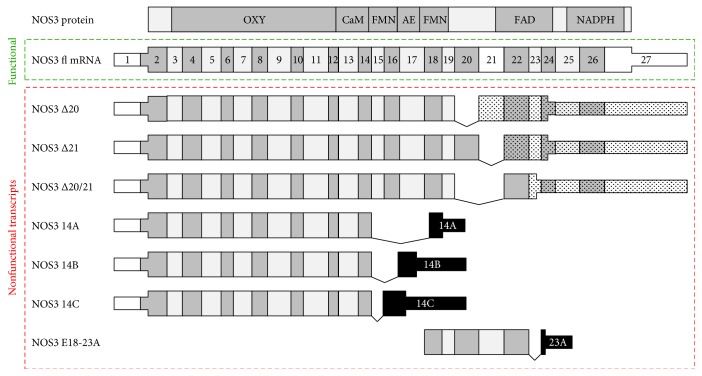
NOS3 transcript isoforms. Depicted at the top is the NOS3 protein (NOS3 protein) with its functional domains (OXY: oxygenase domain; CaM: calmodulin-binding site; FMN: FMN recognition site; AE: autoinhibitory element; FAD: FAD recognition site; NADPH: NADPH recognition site). The corresponding full-length transcript (NOS3 fl mRNA) annotated in Ensembl (v.93) with numbered exons is shown in the green dotted box. The coding region (wide boxes) extends from exon 2 into exon 27. Nonfunctional transcripts, i.e., transcripts not coding for functional NOS3, are shown in the red box below. Skipping of exon 20 (NOS3 *Δ*20), 21 (NOS3 *Δ*21), or both (NOS3 *Δ*20/21) leads to nonfunctional proteins due to frame shifts (dotted boxes) [31]. The variants NOS3 14A/B/C (previously described as NOS3 13A/B/C [32]) originate from splicing events from exon 14 into exons located in the intron of the full-length NOS3 transcript (black boxes). These transcripts terminate in a common polyadenylation signal and encode C-terminally truncated proteins only containing the OXY and CaM domains. The transcript starting at exon 18 (NOS3 E18-23A) lacks the 5′-portion and shows a similar splicing phenomenon as NOS3 14A/B/C at its 3′-end. Thus, it codes for an N- and C-terminally truncated protein.

RNA sequencing data showed that the observed decrease in the NOS3 protein levels was accompanied by a decrease in the total amount of NOS3 mRNA reads. In particular, differential gene expression analysis indicated a significant decrease in the NOS3 RNA levels in high LDL-treated cells compared to the healthy control with an adjusted *p* value of 0.06. However, this analysis did not discriminate functional NOS3 mRNA transcripts from nonfunctional NOS3 transcripts generated by alternative splicing. Therefore, we focused on splice site usage within the NOS3 locus, allowing a more detailed view on protein coding and noncoding transcript isoforms. Indeed, splice site usage analysis showed a reduction in gene normalized splice site usage (GNSSR) for those sites involved in the generation of functional NOS3 mRNA transcripts but an increase in the GNSSR contributing to nonfunctional transcript isoforms (Figure 2 and Table 1).

In this analysis, all exon-exon junctions showing a significant decrease in the relative coverage upon LDL treatment were located upstream of exon 18. In contrast, a significant increase was found in the junctions downstream of exon 18 (Table 1). This reciprocal regulation cannot simply be explained with the full-length transcript but might rather be indicative of aberrant transcription from internal promoters generating transcripts not producing functional NOS3 protein. In both regions, the junctions indicative of alternative splicing from exon 14 into exons 14A/B/C or resulting in skipping of exon 21 represented only a small fraction of the reads. Thus, in addition to the overall decrease in NOS3 reads, the ratio of gapped reads (exon-exon junction reads) assigned to functional and nonfunctional transcript isoforms is altered towards the latter, which provides an adequate explanation for the reduction of functional NOS3 protein. In agreement with previous observations [22], these results additionally demonstrate that splice site usage can be influenced by high LDL. Differences in the extent of splice site usage between healthy and unhealthy conditions are likely to originate from differential gene expression of genes coding for splicing regulatory proteins (Suppl.  Table 1), which mediate splice site usage and consequently RNA transcript isoform levels.

### 3.2. High LDL Reduces Migratory Capacity, Expression of Genes Associated with Cell Migration, and ATP Content

**Figure 3:**
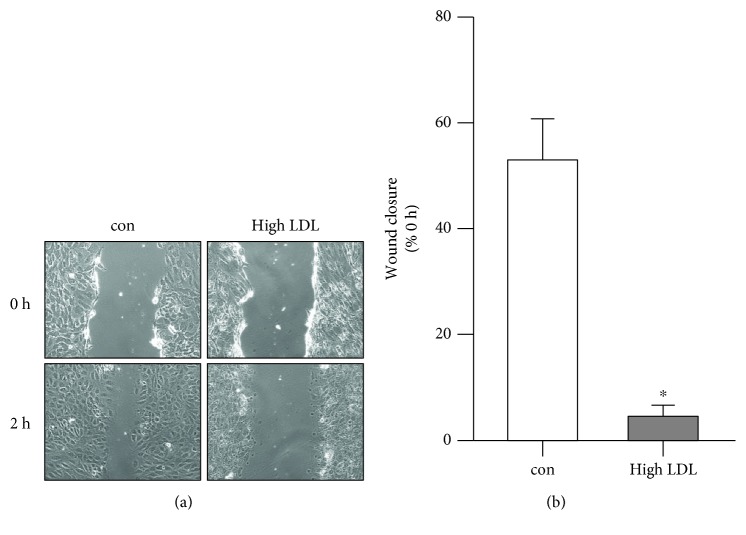
High LDL decreases migratory capacity of endothelial cells. Human primary endothelial cells were cultured in standard medium (con) or medium containing 1 mg/ml LDL (high LDL) for 5 days. A wound was set, and wound width was determined directly afterwards (0 h) and two hours later (2 h). (a) Representative microscopic pictures. (b) Wound closure relative to the 0 h time point. Data are mean ± SEM; *n* = 4; *p* < 0.05 vs. con.

A reduction in the functional NOS3 levels due to an unhealthy treatment leads to a decrease in the NO levels. Since endothelial cell migration is NO-dependent [23–25], we next investigated endothelial cell migration under high LDL conditions. Endothelial cells treated with high LDL were severely impaired to close a wound (Figure 3).

**Figure 4:**
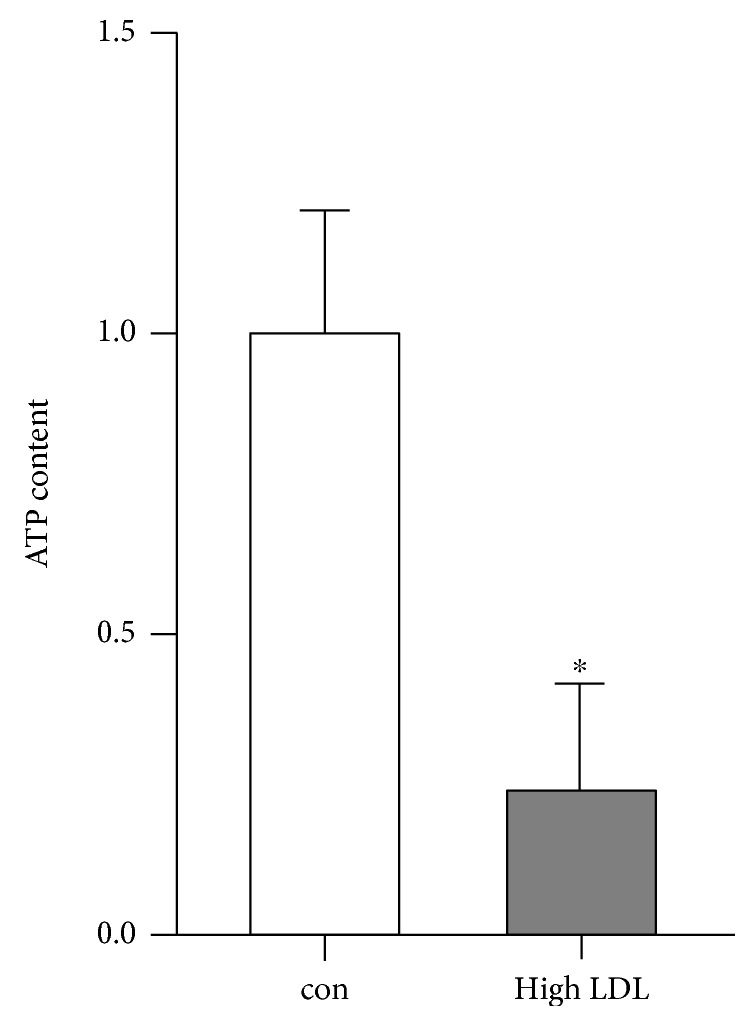
High LDL treatment significantly reduces ATP content in endothelial cells. Human primary endothelial cells were cultured in standard medium (con) or medium containing 1 mg/ml LDL (high LDL) for 5 days, and ATP content was measured. Data are mean ± SEM; *n* = 4; *p* < 0.05 vs. con.

To address the question whether the expression of genes known to be associated with cell migration capacity could substantiate our finding, we performed differential gene expression analysis. Indeed, the expression of the cell cycle controlling protein CDC42, which is involved in cell migration was reduced by 35% in cells under high LDL conditions (*p* < 0.05). Another factor associated with cell migration, AKT1, was also significantly lower expressed by 17% in high LDL-treated cells (*p* < 0.05). Furthermore, in RNA samples of cells incubated for one week with high LDL, Rho family GTPases RND1 and RND3, but not RND2, showed a decrease in their expression by 55% (*p* < 0.05) and 39% (*p* < 0.05), respectively. RND1/2/3 are known to play a role in cell migration [26]. The reduction in CDC42, AKT1, and RND-transcript levels indicated a negative effect of high LDL on the expression of genes, associated with migration of human endothelial cells. Thus, besides the high LDL mediated decrease in the NO levels, also genes associated with cell migration were significantly decreased in their expression providing a plausible explanation for the observed reduced migratory capacity. Since we have previously demonstrated that the migration of primary human endothelial cells depends on intact mitochondria [7, 9], we next determined the ATP content in those cells. Indeed, the ATP levels in cells treated with high LDL were significantly reduced (Figure 4).

The dependence of migratory capacity on the ATP content was further confirmed by treatment of endothelial cells with oligomycin—a specific inhibitor of the mitochondrial ATP synthase. Both migratory capacity and ATP content were decreased by approximately 60% (Suppl.  Figure 1). The latter finding underscores that the mitochondria are one of the main energy sources in endothelial cells.

### 3.3. Effects of High LDL on Mitochondrial DNA and RNA Levels as well as on Mitochondrial Mass

As we found reduced migratory capacity as well as lower ATP content in primary human endothelial cells upon treatment with high LDL, we next investigated the effects of high LDL on mitochondrial DNA and RNA levels, as well as on mitochondrial mass. Therefore, we first performed an alignment of the sequencing data to the human reference genome. In control samples (con), around 95.5% of the total reads could be mapped to the nuclear and 4.5% to the mitochondrial genome (Table 2). However, in high LDL-treated cells, around 11.2% of the reads could be mapped to mitochondrial transcripts. Thus, high LDL led to a significant, more than twofold increase in the mitochondrial RNA (mtRNA) levels.

**Figure 5:**
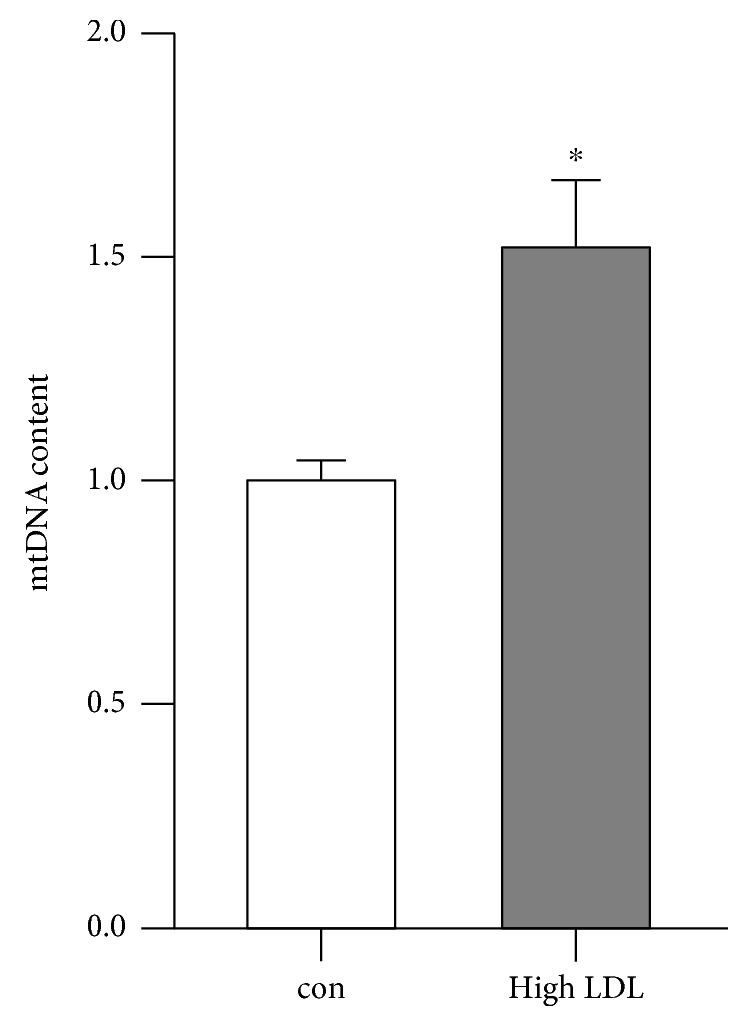
High LDL increases mtDNA content. Human primary endothelial cells were cultured in standard medium (con) or medium containing 1 mg/ml LDL (high LDL) for 7 days. Total DNA was isolated, and mtDNA content was measured by semiquantitative real-time PCR using NXN as nuclear reference gene. Data are mean ± SEM; *n* = 6; *p* < 0.05 vs. con.

The upregulation of mtRNAs upon high LDL treatment raised the question as to whether this is paralleled by an elevation in mitochondrial DNA (mtDNA) content. Corresponding to the increase in mtRNA content, high LDL-treated cells showed a significantly higher mtDNA content (Figure 5).

**Figure 6:**
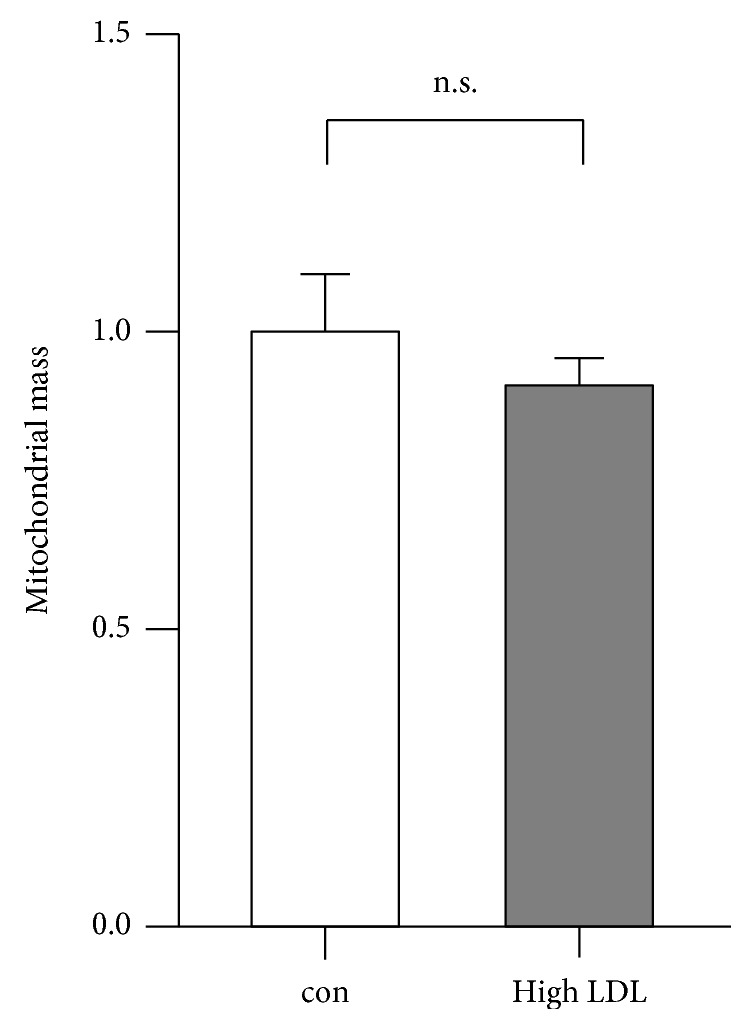
Mitochondrial mass is not altered by high LDL treatment. Human primary endothelial cells were cultured in standard medium (con) or medium containing 1 mg/ml LDL (high LDL) for 7 days. Then, cells were incubated with nonyl acridine orange and analyzed by flow cytometry. Data are mean ± SEM; *n* = 4; n.s. = not significant.

The increase in mtRNAs and mtDNA could be indicative of a higher number in mitochondria. Therefore, we determined the expression of genes coding for the translocases of outer (TOMMs) and inner (TIMMs) mitochondrial membrane proteins as surrogate markers. Those transcripts, however, were either not regulated or expressed at lower levels upon high LDL treatment. Corresponding to the non- or downregulated mRNA transcript levels, analysis of the TIMM23 protein levels as a marker for mitochondria showed no significant difference between the two conditions (data not shown). Thus, an increase in overall mitochondrial number induced by high LDL treatment was rather unlikely. We substantiated this by measuring mitochondrial mass using nonyl acridine orange. As shown in Figure 6, high LDL treatment did not result in a change in mitochondrial mass.

As the mtDNA content was increased upon high LDL without a concomitant change in mitochondrial mass, we next investigated the expression of protein coding mtRNA transcripts and mitochondrial ribosomal RNAs. Therefore, the RNA sequencing data were again analyzed for this specific subset of transcripts. High LDL-treated cells displayed an increase in the expression of these mitochondrial transcripts (Table 3).

**Figure 7:**
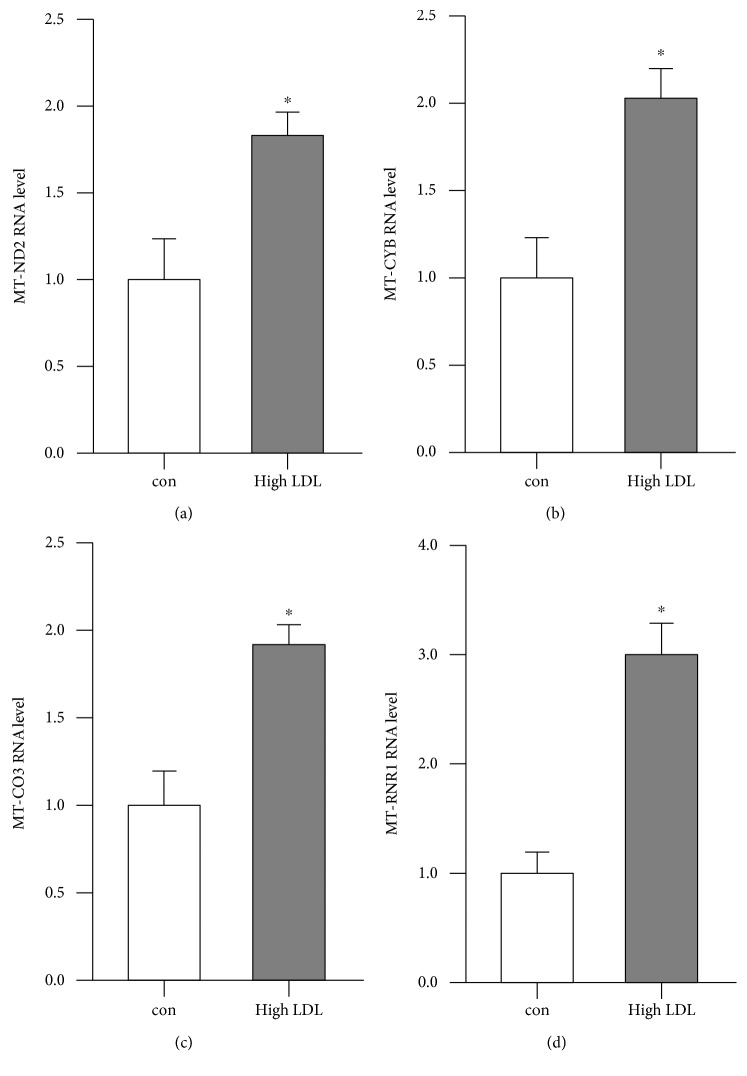
Transcript levels of mitochondrial genes are increased after treatment with high LDL for 7 days. Human primary endothelial cells were cultured in standard medium (con) or medium containing 1 mg/ml LDL (high LDL) for 7 days. Semiquantitative real-time PCRs were performed for MT-ND2 (a), MT-CYB (b), MT-CO3 (c), and MT-RNR1 (d) using RPL32 as reference. Data are mean ± SEM; *n* = 4; *p* < 0.05 vs. con.

To validate our RNA sequencing data, the transcript levels of mitochondrially encoded NADH:ubiquinone oxidoreductase core subunit 2 (MT-ND2), cytochrome B (MT-CYB), cytochrome C oxidase III (MT-CO3), and the mitochondrial 12S RNA (MT-RNR1) were analyzed by real-time PCR. The first three are subunits of electron transport chain complexes I, III, and IV, respectively. All of the chosen transcripts were significantly increased after treatment with high LDL (Figure 7).

**Figure 8:**
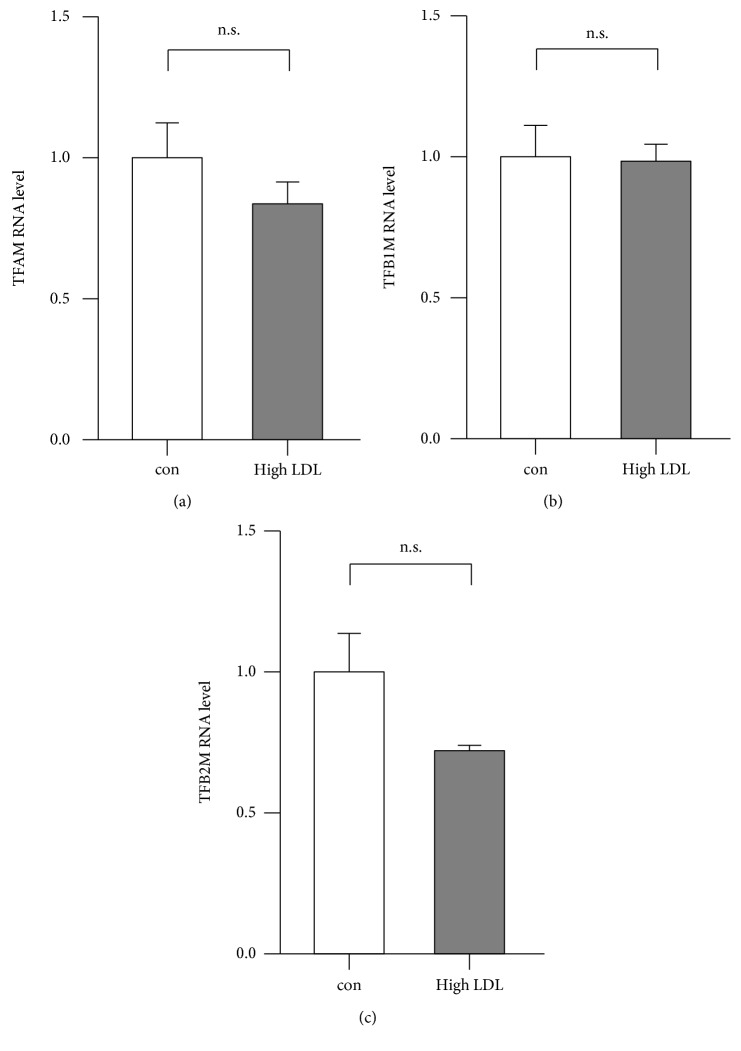
Transcript levels of nuclear-encoded transcription factors of mtDNA transcription are not regulated by high LDL treatment. Human primary endothelial cells were cultured in standard medium (con) or medium containing 1 mg/ml LDL (high LDL) for 7 days. Semiquantitative real-time PCRs were performed for TFAM (a), TFB1M (b), and TFB2M (c) using RPL32 as reference. Data are mean ± SEM; *n* = 4; n.s. = not significant.

Since the mtRNAs were upregulated, we next investigated whether the nuclear-encoded transcription factors, which are known to be mainly responsible for the transcription of mtDNA, are regulated by high LDL. Expression analysis of the transcripts coding for mitochondrial transcription factor A (TFAM), B1 (TFB1M), and B2 (TFB2M) by real-time PCR, however, did not indicate any significant differences (Figure 8), suggesting that the increase in the mtRNA levels is also not related to an increase in transcription.

**Figure 9:**
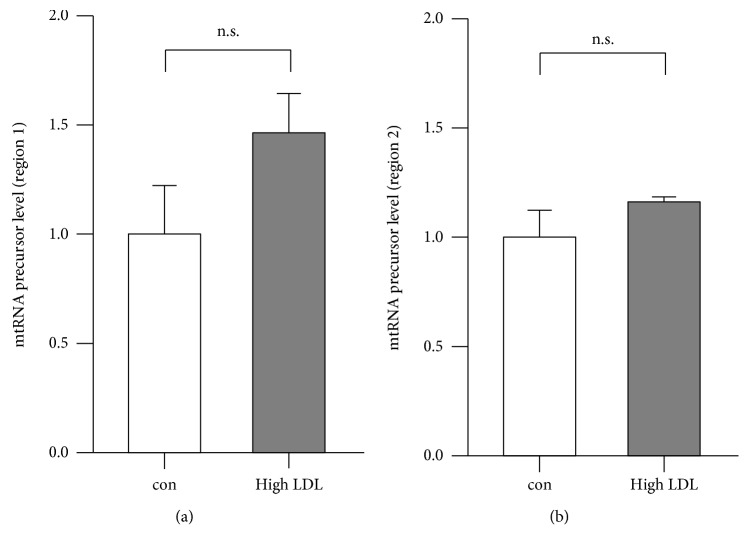
mtRNA precursor transcripts are not regulated by high LDL treatment. Human primary endothelial cells were cultured in standard medium (con) or medium containing 1 mg/ml LDL (high LDL) for 7 days. Semiquantitative real-time PCRs were performed for mtRNA precursor transcripts using RPL32 as reference. Data are mean ± SEM; *n* = 3 − 4; n.s. = not significant.

After transcription, the mtRNA precursor transcripts are cleaved by RNase P and RNase Z at the 5′- and 3′ end, respectively, leading to mature mt-mRNA, mt-rRNA, and mt-tRNA transcripts within the mitochondrial matrix. To exclude that high LDL treatment did not impair processing of the precursor mtRNA transcripts, the actual amount of precursor mtRNA transcripts was estimated by counting reads whose alignment covered the border of two neighboring mtRNA transcripts, which get separated after processing by the RNases. Taking the border coordinates of every mtRNA transcript from the human reference genome GRCh38/hg38 (Ensembl version 91), a combination of every transcript border coordinate of neighboring elements of the mitochondrial genome was generated. For every pair, reads were subsequently counted, which simultaneously covered the downstream end of one transcript and the upstream end of the transcript downstream to it, with a minimal overlap of six nucleotides. For comparison across sample groups, the coverage per border pair was normalized to the total number of ungapped reads in a given sample and the relative amount of ungapped reads mapped to the mitochondrial reference sequence. The average border coverage within a sample was then used as an approximation for the relative amount of mtRNA precursor transcripts. Comparing transcript border coverages did not reveal significant differences in mtRNA precursor transcripts (data not shown). For validation, we performed RT PCRs across borders of mature transcripts, thereby detecting the unprocessed precursors. These experiments corroborated the bioinformatic analyses (Figure 9).

Although some proteins of the respiratory chain are encoded on the mitochondrial DNA, most of them are derived from nuclear genes. Undisturbed interplay of mitochondrial and nuclear-encoded proteins ensures efficient respiratory chain complex formation and consequently ATP synthesis [27]. Since treatment with high LDL led to an increase in the expression of mitochondrial encoded proteins, we also analyzed differential expression of nuclear genes for respiratory chain proteins (Suppl.  Table 2).

In contrast to the overall increased mitochondrial transcript levels following high LDL treatment, differential gene expression analysis for 81 nuclear genes encoding proteins of the respiratory chain revealed that 32% of them were significantly downregulated and only 4% upregulated.

The increase in mitochondrial gene expression and decrease in one third of nuclear genes for respiratory chain proteins could, therefore, restrict efficiency of ATP production due to an imbalance in respiratory chain subunits preventing proper assembly. Thus, one could assume that the reduced ATP content seen in Figure 4 is caused by dysfunctional or not correctly assembled respiratory chain complexes.

## 4. Discussion

The major findings of our study are that treatment of human primary endothelial cells with 1 mg/ml LDL for seven days decreases the NOS3 protein levels, increases inactive NOS3 splice variants, and reduces mitochondrial functionality in endothelial cells, which results in dramatically reduced migratory capacity and thus, endothelial cell impairment.

A functional endothelial cell layer is important, since it not only regulates vascular tone but as a barrier also regulates the nutrition uptake of the surrounding tissue and protects against pathogens. Endothelial cells are in direct contact with the bloodstream and consequently the first cells affected by LDL. Here, we demonstrate that treatment of endothelial cells with high LDL leads to decreased levels of functional NOS3 protein and mRNA levels. Additionally, RNA sequencing analyses revealed an increase in inactive NOS3 splice variants. This is accompanied by an increase in the expression of all mitochondrially encoded transcripts. However, there was no increase in total mitochondria number, as shown at the RNA level as well as at the protein level. It was previously described that NOS3-deficient mice showed a dysfunctional mitochondrial *β*-oxidation [28]. This could lead to an increase in reactive oxygen species (ROS) formation and therefore oxidative stress within the cell. The peroxisome proliferator-activated receptor *γ* (PPARG), which is known to regulate the redox balance, fatty acid oxidation, and mtDNA levels, could therefore be one reason for the high mtDNA levels. Its activation upon oxidative stress could potentially lead to the activation of genes holding a PPAR response element (PPRE) in their promoter resulting in an increase in mtDNA copies [29]. Our RNA sequencing data revealed that cells with low levels of functional NOS3 protein showed an increase in PPARG expression by 110%. The increase in mtRNA transcripts upon high LDL is also in line with findings in mice, which were fed a high-fat, high-sucrose diet for 6 weeks [30] and showed an upregulation of several genes important for mitochondrial biogenesis.

We previously demonstrated that those concentrations of LDL resulted in endothelial cell senescence and increased ROS formation. Thus, we hypothesize that cells try to compensate the increased cellular stress, caused by those unhealthy conditions, by upregulating the expression profile of mitochondrial genes, like MT-ND2 and MT-CO3. Mitochondrial encoded genes are all part of the respiratory chain complexes. Thus, the cells try to cope for energy to handle the unfavorable situation. However, the majority of proteins needed for functional complex formation within the respiratory chain are encoded in the nuclear genome. Our data demonstrate, however, that most of those nuclear-encoded genes are downregulated upon high LDL treatment. Thus, an imbalance in proteins needed for the respiratory chain complexes seems plausible. This in turn would disturb efficient complex formation, resulting in reduced ATP production, which subsequently impairs ATP-dependent processes like endothelial cell migration as we show here.

## 5. Conclusions

We demonstrate that high LDL concentrations lead to low NOS3 levels in primary human endothelial cells, which is paralleled by mitochondrial dysfunction. We found an increase in mtDNA copy number and mtRNA levels as a potential compensatory mechanism for an unfavorable situation. However, due to an expression imbalance between nuclear and mitochondrial encoded proteins of the respiratory chain, complex formation is most likely impaired resulting in a drastic reduction in ATP levels. Consequently, the migratory capacity of the endothelial cells is reduced, which would negatively affect several cardiovascular diseases.

## Figures and Tables

**Table 1:** Relative NOS3 exon-exon junction expression. Comparison of relative expression of all exon-exon junctions from exon 1 to exon 18 (1 : 18), exon 18 to exon 27 (18 : 27), and exon-exon junctions indicative of alternative splicing from exon 14 onto exons 14A/B/C (14-14x) or skipping exon 21 (20-22). The exon-exon junction coverage was normalized per sample to the number of gapped reads within the sample and to gene expression; the *p* value was calculated with Student's *t*-test. Exon-exon junctions indicative of skipping exon 20 or exon 20 and 21 simultaneously were not detectable.

Exon-exon junction	Average normalized expression		*p* value
	con	High LDL	
1 : 18	3,740	3,153	0.00
18:27	3,949	4,876	0.02
14-14x	0.009	0.010	0.83
20-22	0.002	0.024	0.16

**Table 2:** Percentage of read numbers representing nuclear and mitochondrial transcripts. Shown are total read numbers for all individual biological replicates, i.e., RNAs isolated from human primary endothelial cells cultured in standard medium (con_1-4) or medium containing 1 mg/ml LDL (high_LDL-1-4) and the percentage of reads, which could be mapped to the nuclear or mitochondrial reference genome.

Sample	Nuclear (%)	Mitochondrial (%)	Total # of reads
con_1	96.0	4.0	325,725,044
con_2	95.8	4.2	319,428,178
con_3	93.9	6.1	335,842,395
con_4	96.3	3.7	325,882,327
high_LDL_1	88.9	11.0	328,183,964
high_LDL_2	88.1	11.9	327,355,990
high_LDL_3	89.0	11.0	331,832,849
high_LDL_4	89.0	11.0	331,650,314

**Table 3:** Differential gene expression of mitochondrial transcripts after high LDL treatment. Comparison of the expression of mitochondrial protein coding genes and ribosomal RNAs between untreated cells and cells treated with high LDL. The L2FC (log 2-fold change) states the average difference in gene expression between both treatments. Positive L2FC values denote upregulation by high LDL; negative values denote downregulation. A Wald test from DESeq2 was used to calculate the significance of the change in the expression. The adjusted *p* values take the number of tested genes into account.

Gene name	Ensembl gene ID	L2FC	*p* value	Adjusted *p* value
MT-RNR1	ENSG00000211459	1.86	1.46*E* − 40	2.01*E* − 38
MT-RNR2	ENSG00000210082	1.52	1.66*E* − 30	1.41*E* − 28
MT-ND6	ENSG00000198695	1.67	2.98*E* − 24	1.68*E* − 22
MT-ND1	ENSG00000198888	1.34	3.02*E* − 23	1.58*E* − 21
MT-ND4	ENSG00000198886	1.29	9.81*E* − 22	4.50*E* − 20
MT-ND3	ENSG00000198840	1.33	1.48*E* − 20	6.17*E* − 19
MT-CO1	ENSG00000198804	1.34	3.43*E* − 20	1.38*E* − 18
MT-CO2	ENSG00000198712	1.19	4.09*E* − 18	1.36*E* − 16
MT-ATP6	ENSG00000198899	1.00	2.94*E* − 14	6.34*E* − 13
MT-CYB	ENSG00000198727	1.01	9.95*E* − 13	1.76*E* − 11
MT-ND2	ENSG00000198763	0.90	1.46*E* − 11	2.18*E* − 10
MT-CO3	ENSG00000198938	0.87	2.24*E* − 10	2.77*E* − 09
MT-ND4L	ENSG00000212907	1.04	4.25*E* − 10	5.01*E* − 09
MT-ND5	ENSG00000198786	1.40	2.67*E* − 08	2.31*E* − 07
MT-ATP8	ENSG00000228253	0.82	7.33*E* − 08	5.85*E* − 07

## Data Availability

The RNA sequencing data used to support the findings of this study have been deposited at ArrayExpress under accession number E-MTAB-7647. All other data are available upon request.

## Supplementary Materials

Supplementary Materials Supplementary Figure 1: oligomycin reduces migratory capacity and ATP content in primary human endothelial cells. Primary human endothelial cells were left untreated (con) or treated with 10 *μ*M oligomycin for 24 hours, and migratory capacity (A) and ATP content (B) were measured. Data are mean ± SEM; *n* = 4 − 5; *p* < 0.05 vs. con. Supplementary Table 1: differential gene expression of splicing regulatory genes. DGE calculated using the R package DESeq2 of genes encoding splicing regulatory proteins in samples of untreated cells versus cells treated with high LDL for 7 days. Genes significantly varying in expression are marked in bold. The L2FC (log 2-fold change) states the average difference in gene expression between both treatments. Positive L2FC values denote upregulation by high LDL; negative values denote downregulation. A Wald test from DESeq2 was used to calculate the significance of the change in the expression. The adjusted *p* values take the number of tested genes into account. Supplementary Table 2: differential gene expression of nuclear-encoded proteins of the mitochondrial electron transport chain. DGE calculated using the R package DESeq2 of genes encoding proteins of the mitochondrial electron transport chain (ETC) in samples of untreated cells versus cells treated with high LDL for 7 days. Genes significantly varying in expression are marked in bold. The L2FC (log 2-fold change) states the average difference in gene expression between both treatments. Positive L2FC values denote upregulation by high LDL, negative values denote downregulation. A Wald test from DESeq2 was used to calculate the significance of the change in the expression. The adjusted *p* values take the number of tested genes into account.
