# Supplementary material for: High Concentration of Low-Density Lipoprotein Results in Disturbances in Mitochondrial Transcription and Functionality in Endothelial Cells
Source: Oxid Med Cell Longev. 2019 Jun 10;2019:7976382. doi: 10.1155/2019/7976382 (PMC6590621; doi:10.1155/2019/7976382)
Supplement: Supplementary Materials — Supplementary Figure 1: oligomycin reduces migratory capacity and ATP content in primary human endothelial cells. Primary human endothelial cells were left untreated (con) or treated with 10 μM oligomycin for 24 hours, and migratory capacity (A) and ATP content (B) were measured. Data are mean ± SEM; n = 4 − 5; p < 0.05 vs. con. Supplementary Table 1: differential gene expression of splicing regulatory genes. DGE calculated using the R package DESeq2 of genes encoding splicing regulatory proteins in samples of untreated cells versus cells treated with high LDL for 7 days. Genes significantly varying in expression are marked in bold. The L2FC (log 2-fold change) states the average difference in gene expression between both treatments. Positive L2FC values denote upregulation by high LDL; negative values denote downregulation. A Wald test from DESeq2 was used to calculate the significance of the change in the expression. The adjusted p values take the number of tested genes into account. Supplementary Table 2: differential gene expression of nuclear-encoded proteins of the mitochondrial electron transport chain. DGE calculated using the R package DESeq2 of genes encoding proteins of the mitochondrial electron transport chain (ETC) in samples of untreated cells versus cells treated with high LDL for 7 days. Genes significantly varying in expression are marked in bold. The L2FC (log 2-fold change) states the average difference in gene expression between both treatments. Positive L2FC values denote upregulation by high LDL, negative values denote downregulation. A Wald test from DESeq2 was used to calculate the significance of the change in the expression. The adjusted p values take the number of tested genes into account. [file 7976382.f1.pdf]

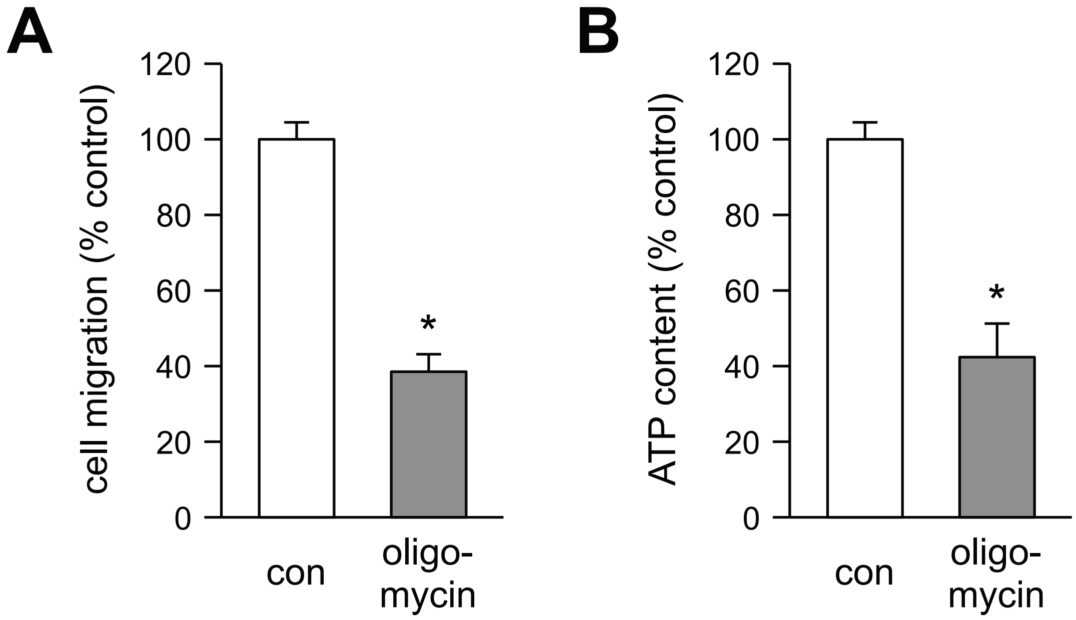

Supplementary Figure 1: Oligomycin reduces migratory capacity and ATP content in primary human endothelial cells. Primary human endothelial cells were left untreated (con) or treated with 10 µM oligomycin for 24 hours and migratory capacity (A) and ATP content (B) were measured. Data are mean ± SEM, n=4-5, $p<0.05$ vs con.

Supplementary Table 1: Differential gene expression of splicing regulatory genes. DGE calculated using the R package DESeq2 of genes encoding splicing regulatory proteins in samples of untreated cells versus cells treated with high LDL for 7 days. Genes significantly varying in expression are marked in bold. The L2FC (Log 2-fold change) states the average difference in gene expression between both treatments. Positive L2FC values denote upregulation by high LDL, negative values downregulation. Wald test from DESeq2 was used to calculate the significance of the change in the expression. The adjusted p-values take the number of tested genes into account.

Gene name	Ensembl gene ID	L2FC	p-value	adj. p-value
**SRSF3**	**ENSG00000112081**	**-0.640**	**0.000**	**0.000**
**SRSF10**	**ENSG00000188529**	**-0.316**	**0.000**	**0.000**
**SRSF9**	**ENSG00000111786**	**-0.314**	**0.000**	**0.001**
**SRSF8**	**ENSG00000263465**	**-0.320**	**0.000**	**0.001**
**SRSF12**	**ENSG00000154548**	**0.978**	**0.002**	**0.007**
**SRSF6**	**ENSG00000124193**	**0.215**	**0.011**	**0.028**
**SRSF1**	**ENSG00000136450**	**-0.173**	**0.014**	**0.035**
SRSF2	ENSG00000161547	-0.173	0.024	0.056
SRSF11	ENSG00000116754	0.128	0.210	0.325
SRSF9P1	ENSG00000214867	-0.160	0.750	0.829
SRSF7	ENSG00000115875	-0.023	0.776	0.847
SRSF5	ENSG00000100650	-0.013	0.813	0.875
SRSF4	ENSG00000116350	0.028	0.825	0.884
**HNRNPLL**	**ENSG00000143889**	**-0.615**	**0.000**	**0.000**
**HNRNPR**	**ENSG00000125944**	**-0.772**	**0.000**	**0.000**
**HNRNPK**	**ENSG00000165119**	**-0.564**	**0.000**	**0.000**
**HNRNPD**	**ENSG00000138668**	**-0.367**	**0.000**	**0.000**
**HNRNPU**	**ENSG00000153187**	**-0.374**	**0.000**	**0.000**
**HNRNPH2**	**ENSG00000126945**	**-0.462**	**0.000**	**0.000**
**HNRNPH3**	**ENSG00000096746**	**-0.384**	**0.000**	**0.000**
**HNRNPUL2-BSCL2**	**ENSG00000234857**	**-0.721**	**0.000**	**0.000**
**HNRNPA3**	**ENSG00000170144**	**-0.416**	**0.000**	**0.000**
**HNRNPA1P16**	**ENSG00000262333**	**0.681**	**0.000**	**0.000**
**HNRNPF**	**ENSG00000169813**	**-0.365**	**0.000**	**0.000**
**HNRNPAB**	**ENSG00000197451**	**-0.343**	**0.000**	**0.000**
**HNRNPA0**	**ENSG00000177733**	**-0.288**	**0.000**	**0.000**
**HNRNPA2B1**	**ENSG00000122566**	**-0.254**	**0.000**	**0.001**
**HNRNPUL1**	**ENSG00000105323**	**-0.267**	**0.001**	**0.003**
**HNRNPCP7**	**ENSG00000228653**	**0.937**	**0.001**	**0.003**
**HNRNPA1P54**	**ENSG00000236539**	**0.801**	**0.001**	**0.004**
**HNRNPA3P9**	**ENSG00000270903**	**-1.260**	**0.002**	**0.006**
**HNRNPUL2**	**ENSG00000214753**	**-0.206**	**0.005**	**0.015**
**HNRNPA1**	**ENSG00000135486**	**-0.167**	**0.012**	**0.031**
HNRNPA1P14	ENSG00000227638	0.802	0.024	0.056
HNRNPH1P1	ENSG00000220305	0.872	0.031	0.069
HNRNPL	ENSG00000104824	-0.162	0.036	0.079
HNRNPA1P49	ENSG00000233231	-0.675	0.043	0.091
HNRNPCP1	ENSG00000258900	0.497	0.054	0.109
HNRNPC	ENSG00000092199	-0.145	0.057	0.115
HNRNPM	ENSG00000099783	-0.172	0.066	0.130
HNRNPH1	ENSG00000169045	-0.180	0.067	0.131
HNRNPA1P3	ENSG00000226188	1.136	0.078	0.148
HNRNPA1L2	ENSG00000139675	0.155	0.099	0.179
HNRNPCP2	ENSG00000204253	0.550	0.138	0.235
HNRNPA1P35	ENSG00000225695	1.065	0.156	0.258
RPL36A-HNRNPH2	ENSG00000257529	-1.110	0.165	0.270
HNRNPA1P48	ENSG00000224578	0.209	0.175	0.283
HNRNPA3P6	ENSG00000213300	-0.577	0.183	0.292
HNRNPA1P53	ENSG00000229534	1.466	0.208	0.323
HNRNPA1P27	ENSG00000233680	0.787	0.210	0.326
HNRNPA1P40	ENSG00000212961	0.468	0.275	0.401
HNRNPA1P59	ENSG00000230280	-0.175	0.315	0.445
HNRNPA1P10	ENSG00000214223	-0.284	0.326	0.455
HNRNPLP1	ENSG00000233503	-0.890	0.331	0.460
HNRNPLP2	ENSG00000259917	0.494	0.500	0.622
HNRNPDL	ENSG00000152795	-0.040	0.558	0.674
HNRNPA3P12	ENSG00000219102	0.102	0.817	0.878
HNRNPA1P7	ENSG00000215492	-0.197	0.859	0.909

Supplementary Table 2: Differential gene expression of nuclear encoded proteins of the mitochondrial electron transport chain. DGE calculated using the R package DESeq2 of genes encoding proteins of the mitochondrial electron transport chain (ETC) in samples of untreated cells versus cells treated with high LDL for 7 days. Genes significantly varying in expression are marked in bold. The L2FC (Log 2-fold change) states the average difference in gene expression between both treatments. Positive L2FC values denote upregulation by high LDL, negative values downregulation. Wald test from DESeq2 was used to calculate the significance of the change in the expression. The adjusted p-values take the number of tested genes into account.

Gene name	Ensembl gene ID	L2FC	p-value	adj. p-value	ETC complex
**NDUFS1**	**ENSG00000023228**	**-0.700**	**0.000**	**0.000**	**complex I**
**NDUFB5**	**ENSG00000136521**	**-0.542**	**0.000**	**0.000**	**complex I**
**NDUFA7**	**ENSG00000267855**	**-0.398**	**0.000**	**0.000**	**complex I**
**NDUFV1**	**ENSG00000167792**	**0.432**	**0.000**	**0.000**	**complex I**
**NDUFV3**	**ENSG00000160194**	**-0.276**	**0.000**	**0.000**	**complex I**
**NDUFA5**	**ENSG00000128609**	**-0.249**	**0.000**	**0.001**	**complex I**
**NDUFA9**	**ENSG00000139180**	**-0.335**	**0.000**	**0.001**	**complex I**
**NDUFB3**	**ENSG00000119013**	**-0.343**	**0.001**	**0.003**	**complex I**
**NDUFB6**	**ENSG00000165264**	**-0.343**	**0.001**	**0.003**	**complex I**
**NDUFC1**	**ENSG00000109390**	**-0.332**	**0.001**	**0.005**	**complex I**
**NDUFS3**	**ENSG00000213619**	**0.332**	**0.002**	**0.006**	**complex I**
**NDUFS4**	**ENSG00000164258**	**-0.236**	**0.004**	**0.012**	**complex I**
NDUFA2	ENSG00000131495	-0.162	0.062	0.122	complex I
NDUFA12	ENSG00000184752	-0.202	0.088	0.163	complex I
NDUFA10	ENSG00000130414	-0.118	0.129	0.223	complex I
NDUFC2	ENSG00000151366	-0.148	0.136	0.232	complex I
NDUFA8	ENSG00000119421	0.138	0.151	0.252	complex I
NDUFB4	ENSG00000065518	-0.096	0.157	0.259	complex I
NDUFA6	ENSG00000184983	-0.139	0.164	0.269	complex I
NDUFS5	ENSG00000168653	-0.157	0.182	0.291	complex I
NDUFB1	ENSG00000183648	-0.144	0.185	0.294	complex I
NDUFB9	ENSG00000147684	-0.141	0.185	0.294	complex I
NDUFB2	ENSG00000090266	-0.165	0.188	0.299	complex I
NDUFS8	ENSG00000110717	0.162	0.189	0.299	complex I
NDUFA1	ENSG00000125356	-0.129	0.250	0.373	complex I
NDUFV2	ENSG00000178127	0.150	0.256	0.379	complex I
NDUFS7	ENSG00000115286	-0.126	0.302	0.430	complex I
NDUFAB1	ENSG00000004779	-0.100	0.414	0.543	complex I
NDUFB7	ENSG00000099795	-0.073	0.503	0.624	complex I
NDUFB10	ENSG00000140990	-0.066	0.519	0.639	complex I
NDUFS2	ENSG00000158864	0.045	0.586	0.698	complex I
NDUFB8	ENSG00000166136	-0.052	0.645	0.747	complex I
NDUFA13	ENSG00000186010	0.035	0.764	0.838	complex I
NDUFA11	ENSG00000174886	-0.026	0.826	0.885	complex I
NDUFA3	ENSG00000170906	0.020	0.856	0.906	complex I
NDUFS6	ENSG00000145494	0.022	0.864	0.912	complex I
NDUFB11	ENSG00000147123	0.018	0.880	0.922	complex I
**SDHD**	**ENSG00000204370**	**-0.680**	**0.000**	**0.000**	**complex II**
**SDHC**	**ENSG00000143252**	**-0.610**	**0.000**	**0.000**	**complex II**
**SDHB**	**ENSG00000117118**	**-0.249**	**0.003**	**0.009**	**complex II**
**SDHA**	**ENSG00000073578**	**-0.004**	**0.971**	**0.981**	**complex II**
**UQCRC2**	**ENSG00000140740**	**-0.197**	**0.001**	**0.002**	**complex III**
**UQCRC1**	**ENSG00000010256**	**-0.241**	**0.007**	**0.020**	**complex III**
**UQCRFS1**	**ENSG00000169021**	**-0.241**	**0.014**	**0.035**	**complex III**
CYC1	ENSG00000179091	-0.226	0.034	0.074	complex III
UQCR10	ENSG00000184076	-0.168	0.141	0.239	complex III
UQCR11	ENSG00000127540	-0.138	0.279	0.406	complex III
UQCRH	ENSG00000173660	-0.110	0.310	0.439	complex III
UQCRQ	ENSG00000164405	-0.039	0.755	0.832	complex III
UQCRB	ENSG00000156467	-0.021	0.834	0.891	complex III
**COX7B**	**ENSG00000131174**	**-0.354**	**0.000**	**0.002**	**complex IV**
**COX8A**	**ENSG00000176340**	**-0.286**	**0.013**	**0.033**	**complex IV**
**COX6A1**	**ENSG00000111775**	**-0.198**	**0.017**	**0.042**	**complex IV**
COX4I1	ENSG00000131143	0.203	0.071	0.137	complex IV
COX7A2	ENSG00000112695	-0.184	0.111	0.197	complex IV
COX6B2	ENSG00000160471	0.509	0.118	0.206	complex IV
COX6C	ENSG00000164919	-0.134	0.191	0.302	complex IV

COX5B	ENSG00000135940	-0.101	0.382	0.511	complex IV
COX7A1	ENSG00000161281	-0.091	0.545	0.663	complex IV
COX5A	ENSG00000178741	-0.027	0.813	0.875	complex IV
COX7C	ENSG00000127184	-0.011	0.914	0.945	complex IV
COX6B1	ENSG00000126267	-0.004	0.978	0.987	complex IV
COX8C	ENSG00000187581	1.957	0.196	NA	complex IV
**ATP5PB**	**ENSG00000116459**	**-0.431**	**0.000**	**0.000**	**complex V (ATPase)**
**ATP5MC3**	**ENSG00000154518**	**-0.397**	**0.000**	**0.000**	**complex V (ATPase)**
**ATP5F1A**	**ENSG00000152234**	**-0.329**	**0.000**	**0.000**	**complex V (ATPase)**
**ATP5MC1**	**ENSG00000159199**	**-0.469**	**0.000**	**0.000**	**complex V (ATPase)**
**ATP5MF**	**ENSG00000241468**	**-0.306**	**0.004**	**0.012**	**complex V (ATPase)**
**ATP5F1D**	**ENSG00000099624**	**0.353**	**0.006**	**0.017**	**complex V (ATPase)**
ATP5PD	ENSG00000167863	-0.169	0.074	0.142	complex V (ATPase)
ATP5PF	ENSG00000154723	-0.182	0.111	0.197	complex V (ATPase)
ATP5F1C	ENSG00000165629	-0.095	0.204	0.319	complex V (ATPase)
ATP5F1B	ENSG00000110955	-0.133	0.213	0.329	complex V (ATPase)
ATP5MD	ENSG00000173915	-0.130	0.253	0.375	complex V (ATPase)
ATP5IF1	ENSG00000130770	-0.111	0.287	0.414	complex V (ATPase)
ATP5MG	ENSG00000167283	-0.079	0.428	0.555	complex V (ATPase)
ATP5F1E	ENSG00000124172	-0.087	0.445	0.572	complex V (ATPase)
ATP5MPL	ENSG00000156411	0.033	0.788	0.856	complex V (ATPase)
ATP5ME	ENSG00000169020	0.029	0.807	0.871	complex V (ATPase)
ATP5PO	ENSG00000241837	-0.021	0.851	0.903	complex V (ATPase)
ATP5MC2	ENSG00000135390	-0.005	0.965	0.978	complex V (ATPase)
